# Enhanced Recyclable Magnetized Palm Shell Waste-Based Powdered Activated Carbon for the Removal of Ibuprofen: Insights for Kinetics and Mechanisms

**DOI:** 10.1371/journal.pone.0141013

**Published:** 2015-10-23

**Authors:** Kien Tiek Wong, Yeomin Yoon, Min Jang

**Affiliations:** 1 Department of Civil Engineering, Faculty of Engineering, University of Malaya, Kuala Lumpur, Malaysia; 2 Department of Civil and Environmental Engineering, University of South Carolina, Columbia, United States of America; 3 Nanotechnology and Catalysis Research Centre (NANOCAT), University of Malaya, Kuala Lumpur, Malaysia; Institute for Materials Science, GERMANY

## Abstract

A novel preparation method of magnetized palm shell waste-based powdered activated carbon (MPPAC, avg. size 112 μm) was developed. The prepared MPPAC was assessed by several physicochemical analyses, and batch tests were performed for ibuprofen (IBP) removal. Field emission scanning electron microscopy (FESEM) and N_2_ gas isotherms revealed that magnetite and maghemite were homogeneous and deposited mostly on the surface of PPAC without a significant clogging effect on the micropores. Isotherm results showed that 3.8% Fe (w/w) impregnated PPAC [MPPAC-Fe(3.8%)] had about 2.2-fold higher maximum sorption capacity (157.3 mg g^-1^) and a 2.5-fold higher sorption density (0.23 mg m^-2^) than pristine PPAC. Both Fourier-transform infrared spectroscopy (FTIR) and isotherm data indicated that the high sorption capacity and density of IBP by MPPAC was primarily attributable to donor-acceptor complexes with the C = O group and dispersive π-π interactions with the carbon surface. Based on kinetic and repeated adsorption tests, pore diffusion was the rate-limiting step, and MPPAC-Fe(3.8%) had about 1.9~2.8- and 9.1~15.8-fold higher rate constants than MPPAC-Fe(8.6%) and palm shell-waste granular activated carbon (PGAC, avg. size 621 μm), respectively. MPPAC showed almost eight fold greater re-adsorption capacity than PPAC due to a thermal catalytic effect of magnetite/maghemite.

## Introduction

With more than 65 million chemicals and formulations currently available commercially, the aquatic environment is often contaminated with a wide variety of organic constituents [1]. Of particular concern are pharmaceuticals; they are continually introduced to the environment through wastewater outfalls and have been found in drinking water [2–4]. Due to the high degree of chemical property diversity, conventional treatment processes, such as biological degradation, chlorine oxidation, coagulation, flocculation, and sedimentation, are often ineffective in removing pharmaceuticals [5]. Thus, the occurrence of pharmaceuticals in water resources reported in surveys conducted in several countries [6, 7] is stimulating the need for water treatment processes that are highly effective, relatively easy to operate, and energy efficient [8, 9].

Numerous studies have been performed to remove pharmaceuticals using membrane filtration (nanofiltration and reverse osmosis), photocatalysis [10], sonolysis [11], oxidation using ozone [12, 13], and electrochemical degradation [14]. Nevertheless, drawbacks, such as toxic transformation products, incomplete removal, very high capital and operation costs, and sophisticated maintenance and skilled personnel requirements have become hurdles to the implementation of these technologies. Additionally, oxidative and biological processes often transform substances from one form to another that may not result in detoxification [15]. Among water treatment technologies, adsorption is used frequently because of the ease of operation, simplicity of design, potential for media regeneration, effectiveness in contaminant attenuation, and absence of transformation products. Soil minerals, activated carbons (ACs), mesoporous silicas, zeolites, and other materials have been studied for their characteristics and adsorption mechanisms [16–19]. Of the adsorbents, AC is used most widely for the removal of organic constituents in water. Furthermore, the production of inexpensive AC from natural materials, such as municipal solid waste and agricultural by-products, has received much attention because it can lead to economical water treatments [18, 20, 21].

In this research, we developed a novel synthesis route to prepare magnetized palm shell-waste based powdered activated carbon (MPPAC), based on a cheap and abundant agriculture carbonaceous waste in many tropical countries. Theoretically, MPPAC could have a lower sorption capacity than non-impregnated PPAC because of significant pore blocking by the nano-magnetite impregnation. To reduce micropore blockage, we attempted to coat magnetic materials primarily on the surface of PPAC. In particular, the preparation procedure developed can produce large quantities of MPPAC in an energy-efficient and cost-effective manner. To our knowledge, this is the first reported study to develop a preparation route of magnetized activated carbon for the application of water treatment.

As a model pharmaceutical pollutant, ibuprofen (IBP) was chosen because it is one of the most widely consumed medicines worldwide, and has been found in many water sources, as well as in waste water [22, 23]. The physical and chemical characteristics of IBP are listed in Table A in S1 File.

The main objectives of this investigation were to (i) prepare MPPAC coated with various amounts of Fe, (ii) characterize the MPPACs using various spectroscopic analysis, (iii) assess the sorption capacities and rates of IBP in batch and repeated operations, (iv) determine the thermodynamic parameters and adsorption mechanism, and finally (v) to assess the regeneration and re-adsorption capability of MPPACs by low thermal treatment.

## Materials and Methods

Commercial PPAC (mesh size 100 × 200, avg. 112 μm) as a solid (pH 9.8) activated by KOH was purchased from Bravo Green Sdn. Bhd., Malaysia. Ferrous sulfate heptahydrate (FeSO_4_·7H_2_O), sodium hydroxide (NaOH), potassium nitrate (KNO_3_), methanol (HPLC grade), and sodium chloride (NaCl) were purchased from R&M Chemical. For the adsorption, IBP (99%) was obtained from Sigma-Aldrich.

### Preparation of MPPACs

Different amounts of FeSO_4_·7H_2_O (27 g, 54 g, or 72 g) were dissolved in 200 mL of distilled water, and 50 g PPAC were added. This suspension was heated at 353 K for 2 h. Then, 50 mL of alkaline solution were prepared using 2.25 g of potassium nitrate (KNO_3_) and 15 g of NaOH. This alkaline solution was added drop-wise into the PPAC suspension under constant stirring. The suspension was then sonicated for 1 h at 353 K. The applied power and frequency of the ultrasound irradiation were 500 W and 50–60 Hz, respectively. The suspension was kept overnight for aging, before washing and drying. The precipitate was washed thoroughly with distilled water to remove chemical residue.

### Determination of Fe content in MPPACs

The amount of iron loaded into the PPAC was determined by an aqua regia method, in which a strong acid mixture was prepared by the combination of 10 mL of nitric acid with 30 mL of hydrochloric acid. MPPAC (0.1 g) was then added to 10 mL of this mixture, and the suspension sonicated for 30 min. Then, 5 mL of the suspension were diluted with distilled water to 50 mL. The suspension was filtered (0.45 μm pore size) and analyzed using inductively coupled plasma mass spectrometry (ICP-MS). Aqua regia extraction results showed 3.8, 7.8, or 8.6% Fe loading on PPAC with 27, 54, or 72 g FeSO_4_·7H_2_O addition, respectively. Thus, these media are referred to as MPPAC-Fe(3.8%), -Fe(7.8%), and -Fe(8.6%), respectively.

### Physicochemical characterization

The prepared media were analyzed by X-ray diffraction (XRD), field emission scanning electron microscopy and energy dispersive X-ray spectroscopy (FESEM/EDS), N_2_ gas isotherms, Fourier-transform infrared (FTIR), and pH_pzc_. An explanation of these analyses is provided in S1 File.

### Batch tests of kinetics and isotherms

All kinetic tests were conducted at pH 7 and room temperature at various ionic strengths. Different MPPACs (8 mg) were used to treat 20 mg L^-1^ IBP solution (100 mL) with ionic strength of 0, 0.1 and 0.5 M. IBP samples were collected at different time intervals over 6 h, and concentrations were subsequently determined using a UV spectrophotometer (Spectroquant Phoro 100, Merck) at 220 nm. The experimental kinetics data were fitted using a pseudo-second order kinetic model (see SI). Adsorption isotherms of IBP for the various MPPACs were investigated using different masses of MPPAC (8–400 mg), temperatures (298, 308, or 318 K), and pH (4 and 7) at 0.1 M of NaCl. For isotherms, the IBP solution applied and the operational conditions of the suspension were the same as for the kinetics experiments. More information about the Langmuir and Freundlich isotherm models are noted in the SI. The isosteric Gibbs free energy, ΔG° of adsorption of IBP onto MPPAC-Fe(3.8%) was calculated using the results of the isotherm and the following equation:

$$Ln(k_{d})=Ln\left(\frac{C_{a}}{C_{e}}\right)=-\frac{\Delta G{}^{\circ}}{RT}$$ (1)

where K_d_ is the equilibrium constant, C_a_ is the amount of IBP adsorbed at equilibrium (mg L^-1^), and C_e_ is the concentration of IBP remaining in the solution at equilibrium (mg L^-1^), T is the solution temperature (K), and R is the ideal gas constant. The isosteric enthalpy ΔH° and entropy ΔS° of adsorption were also calculated from the slope and the intercept of the plot of ln(K_d_) versus 1/T, respectively, using the equation: ΔG° = ΔH° - TΔS°. Thermodynamic parameters were calculated for the adsorption of IBP on MPPAC-Fe(3.8%) at a constant adsorption uptake equal to 50 mg g^-1^ and at pH 7.

### Repeated adsorption tests

Two sets of repeated adsorption tests were conducted. In the first trial, MPPAC-Fe(3.8%), MPPAC-Fe(8.6%), and palm shell-waste-based granular activated carbon (PGAC, 20 × 40 mesh) were also used to compare the adsorption speeds. Four cycles of experiments were conducted at pH 7 with 5 mg L^-1^ IBP and 0.1 M ionic strength. In the second trial, 13 cycles of repeated adsorption were conducted using MPPAC-Fe(3.8%) with the same conditions described in the first trial, but with a shortened operational time. MPPAC media were separated from the suspension using a permanent magnet once adsorption was complete, whereas PGAC was separated using a membrane filter (Whatman no. 1, 0.45 μm pore size).

### Regeneration and re-adsorption

Adsorption was conducted by adding 0.01 g of medium [PPAC, MPPAC-Fe(3.8%), or MPPAC-Fe(8.6%)] into 30 mg g^-1^ IBP (100 mL) for 24 h. Then, the recovered medium was washed to remove excess IBP and was dried in the oven at 353 K. Six repeated thermal regenerations of the saturated medium were conducted using a muffle furnace (Dae Heung Science, DF-2) for 3 h at 623 K.

## Results and Discussion

### Characterization

**Fig 1:**
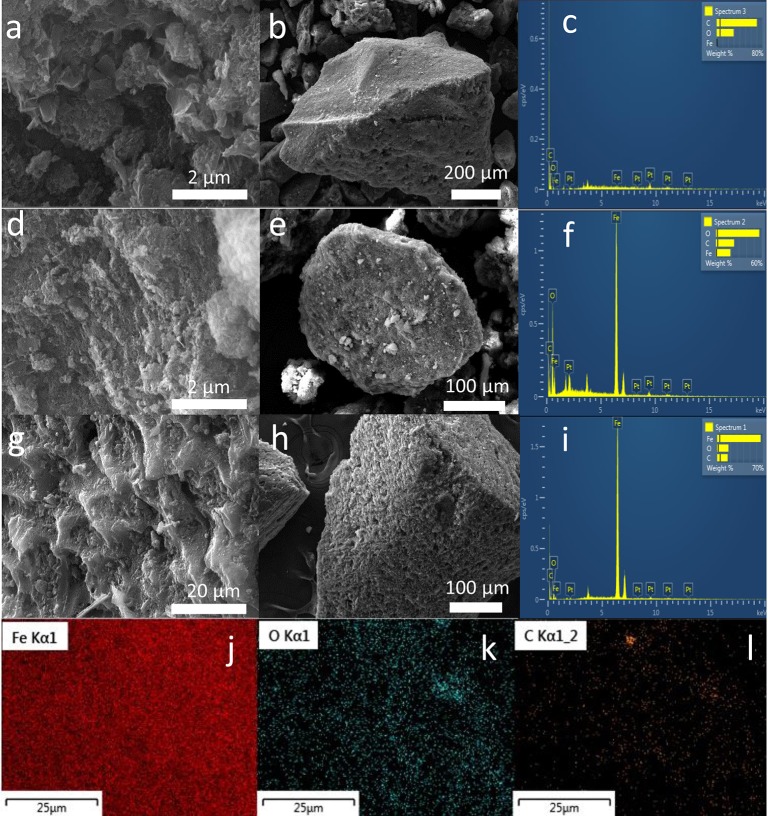
FESEM and EDS images for (a-c) PPAC, (d-f) MPPAC-Fe(3.8%), (g-i) MPPAC-Fe(8.6%), and elemental mapping of (j) Fe, (k) O and (l) C for MPPAC-Fe(8.6%).

The FESEM micrographs and EDS data for PPAC and MPPACs coated with 3.6% and 8.6% Fe are shown in Fig 1A–1I. A rough surface texture of PPAC is seen in Fig 1A. After iron impregnation [Fig 1D and 1G], the smooth surface of PPAC were occupied, and the spongy texture were observed. EDS results, shown in Fig 1C, 1F and 1I, indicate that PPAC did not contain any Fe, while MPPAC-Fe(3.6%) and -Fe(8.6%) had 19.1 and 66.5 wt%, respectively. In particular, the homogeneous dispersion of Fe in MPPAC-Fe(8.6%) observed from elemental mapping indicated that US irradiation was a reliable technique for coating of Fe on the surface of PPAC (Fig 1J).

**Fig 2:**
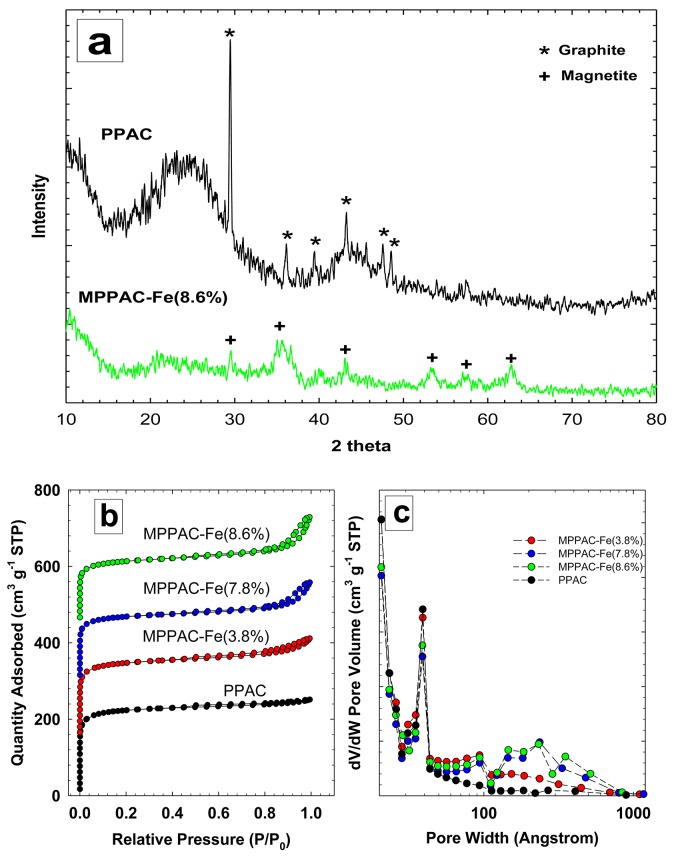
(a) XRD pattern for PPAC and MPPAC−Fe(8.6%), (b) N_2_ gas adsorption–desorption isotherms and (c) differential pore volume according to pore width calculated by Halsey equation with FAAS correction.

Fig 2A shows XRD patterns of PPAC and MPPAC-Fe(8.6%). The XRD patterns of PPAC indicated a typical amorphous carbon shape and showed broad asymmetric peaks, corresponding from 25° to ~45° [24]. Additionally, the pattern showed one peak at ~30°, corresponding to the (4 0 0) reflexes of graphite (JCPDS File, No. 1–640). The XRD analysis of MPPAC-Fe(8.6%) showed that the medium contained two different phases of iron oxide: magnetite at 35.42° (3 1 1) and maghemite at 43.47° (4 0 0), 53.88° (4 2 2), and 57.16° (5 1 1) (JCPDS file, No. 19–0629). The intensity of the graphite peak was reduced sharply in MPPAC-Fe(8.6%) due to the incorporation of magnetite and maghemite.

The N_2_ adsorption-desorption isotherms of all media [Fig 2B] indicated a type I isotherm character, based on the IUPAC classification. This result indicated that all materials had relatively high numbers of micropores. Specifically, as shown for PPAC, the marked knee at the low relative pressure and a very low slope in the multilayer (0.8 < P/P_0_ < 1.0) specified a low external surface and the absence of significant mesoporosity. According to the IUPAC classification, all media possessed a H4-type hysteresis loop, displaying a slit-shaped pore characteristic where both adsorption and desorption branches are parallel [25]. As the amount of impregnated Fe increased, however, the graph displayed type IV character isotherms at a high relative pressure (0.8 < P/P_0_ < 1.0), indicating the formation of meso- and macro-pore structures. The Halsey equation with FAAS correction was used to describe differential pore volume according to pore size [20–2000 Å; Fig 2C]. Although all media had a similar primary peak at 39 Å, they had markedly different trends in pore volume at > 40 Å. MPPAC-Fe(3.8%) had a higher pore volume at < 100 Å than the other media, while higher Fe content media had a higher pore volume at > 100 Å. Based on this result, it can be concluded that more-polymerized structures could create higher inter-particle spaces between magnetite and maghemite as the Fe content increased, leading to higher pore volume at > 100 Å.

Detailed pore characteristics of all materials are summarized in Table 1. Among the media, PPAC had the highest total (776.5 m^2^ g^-1^), BET (754.8 m^2^ g^-1^), and micropore surface area (603.7 m^2^ g^-1^). The pore properties, such as total, BET, and micropore surface area and volume, decreased linearly as the Fe amounts incorporated increased, while total and BJH pore volume increased with amount of Fe incorporated (Fig A in S1 File).

**Table 1:** Pore characteristics of PPAC and MPPACs.

Sample	PPAC	MPPAC-Fe(3.8%)	MPPAC-Fe(7.8%)	MPPAC-Fe(8.6%)
Total surface area^a^ (m^2^ g^-1^)	776.5	684.9	585.0	568.1
BET surface area (m^2^ g^-1^)	754.8	666.5	571.5	555.5
Micropore surface area (m^2^ g^-1^)	603.7 (77.7%^b^)	503.2 (73.5%)	446.3 (76.3%)	408.2 (71.9%)
BJH surface area^c^ (m^2^ g^-1^)	88.9 (11.4%)	111.2 (16.2%)	96.2 (16.4%)	109.1 (19.2%)
Total pore volume (cm^3^ g^-1^)	0.39	0.40	0.40	0.43
Micropore volume^d^ (cm^3^ g^-1^)	0.28	0.23	0.20	0.19
BJH pore volume^e^ (cm^3^ g^-1^)	0.084 (21.5%)	0.150 (37.5%)	0.183 (45.8%)	0.229 (53.3%)
pH_PZC_	9.68	NM ^f^	NM	9.11

^a^ Single point surface area at p/p° = 0.201775532.

^b^ Portions of specific surface area [(specific surface area/total surface area)×100%].

^c,d^ BJH desorption cumulative volume of pores between 17.000 Å and 3,000.000 Å width.

^e^ Single point adsorption at p/p° = 0.995.

^f^ not measured.

**Fig 3:**
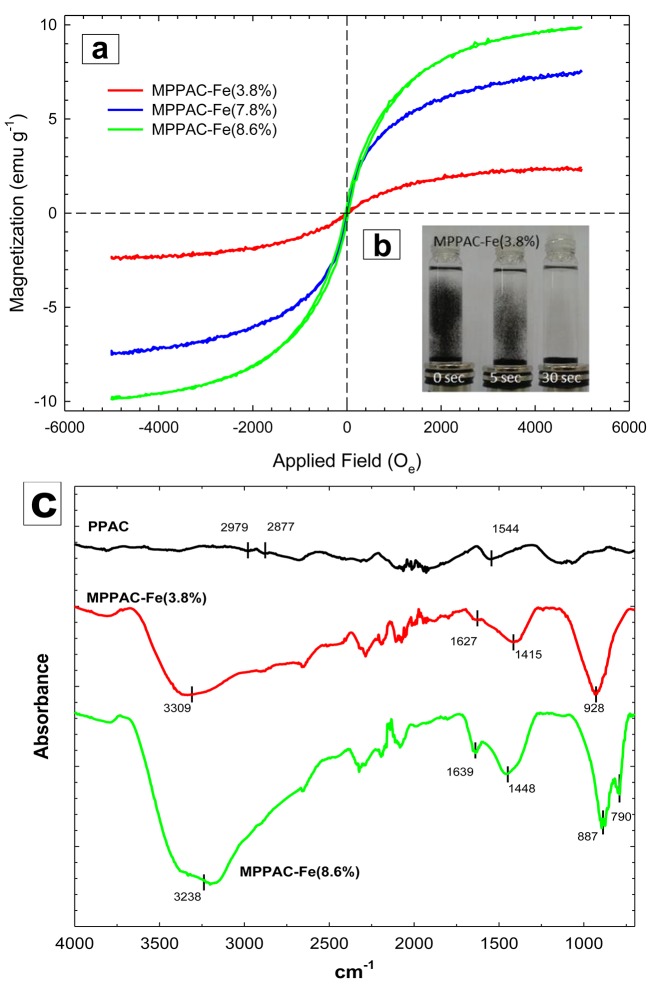
(a) Magnetization versus applied magnetic field for all MPPAC materials and (b) shows settling time of the MPPAC from suspension by external magnet, (c) FTIR spectrum for PPAC, MPPAC–Fe(3.8%) and–Fe(8.6%).

The magnetization of media impregnated with different percentages of Fe was measured at 300 K [Fig 3A]. All media had non-linear and reversible behaviors with magnetic hysteresis loops. As an important magnetic parameter, the saturation magnetization (M_s_) of MPPAC-Fe(3.8%), -Fe(7.8%), and -Fe(8.6%) were 2.3, 6.2, and 9.8 emu g^-1^, respectively. Thus, the magnetic saturation increased as the amount of Fe coated on the PPAC increased. This magnetic property can facilitate ready separation of the media from the suspension by an external magnetic field. Fig 3B shows an illustration of the separation phenomena of MPPAC-Fe(3.8%) with a nickel-plated neodymium magnet (1.25 T). The complete separation took ~30 s.

FTIR analysis was conducted to detect the presence of various organic groups on the surface of PPAC, MPPAC-Fe(3.8%), and -Fe(8.6%) [Fig 3C]. Two wide bands of the hydroxyl (OH^-^) group at 3238 and 3309 cm^-1^ were seen for both MPPAC-Fe(3.8%) and -Fe(8.6%), while they were hardly seen for PPAC. Instead, PPAC had two peaks at 2979 and 2877 cm^-1^, indicating the presence of CH stretching [26]. Additionally, a peak at 1544 cm^-1^ in the IR spectrum of PPAC indicated C = C of an aromatic ring [27]. When Fe was impregnated, the C = C aromatic signal disappeared, but carbonyl (C = O) and phenolic groups were found at 1627/1639 and 1415/1448 cm^-1^ for 3.8/8.6% Fe-impregnated media [28]. Also, the 3.8% Fe-impregnated medium had a smaller C = O signal than 8.6%. Based on these results, it can be concluded that the Fe impregnation using ultrasound resulted in oxidation of the C = C group, forming phenolic and C = O groups, although the degree of oxidation differed. Sharp peaks at 928 and 887 cm^-1^ were found for 3.8 and 8.6% Fe-impregnated media, while PPAC did not show those peaks. These peaks correspond to the intrinsic stretching vibration of Fe at a tetrahedral site. Furthermore, MPPAC-Fe(8.6%) had a peak at 790 cm^-1^, assigned to an octahedral Fe-stretching vibration [28].

### Kinetics with various ionic strength and pH

**Fig 4:**
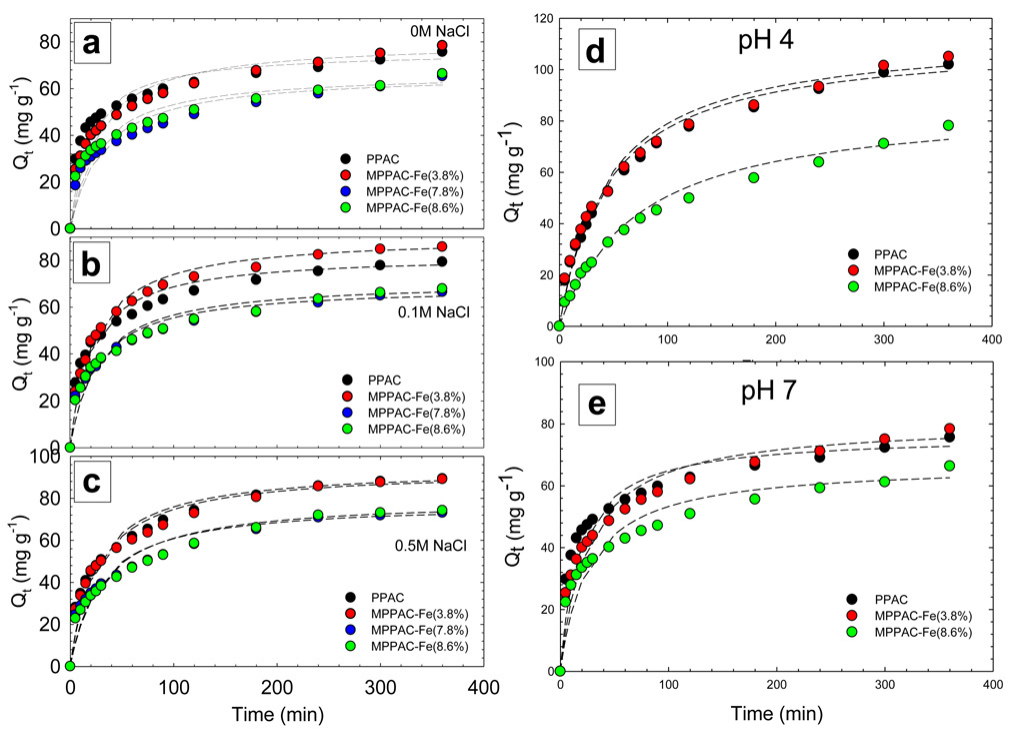
(a-c) Adsorption kinetics of IBP by various media at pH 7.0 at different ionic strength (0 ~ 0.5 M) (fit lines obtained from Pseudo-second order kinetic model), kinetics of IBP uptake by media at pH 4 (d) and 7 (e) at 0.1 M of ionic strength.

(Fig 4A–4C) shows the kinetics of IBP uptake by PPAC or MPPACs in different ionic strengths at pH 7.0. All media showed rapid sorption rates initially, during the first 100 min, and pseudo-equilibrium was reached in 200 min. The rapid initial adsorption may present a more accessible surface as well as high availability of active sites, whereas the observed plateau corresponds to a slower rate of adsorption due to the accumulation of IBP on the active sites. At 0 M ionic strength, PPAC had the highest initial sorption rate for 50 min, while MPPAC-Fe(3.8%) had a higher sorption speed and capacity than other media at 0.1 M ionic strength. In all cases, the sorption capacities and rates of MPPAC-Fe(7.8%) and -Fe(8.6%) were similar, but lower than that of MPPAC-Fe(3.8%).

Through a pseudo-second order kinetic model, the experimental data showed determination coefficients (R^2^) higher than 0.99, indicating reliable fitting. The data obtained for each medium, in terms of values of q_eq_, k_2_, and v_0_, are compared in Table B in S1 File. The equilibrated adsorption capacities increased for all media as the ionic strength increased from 0 to 0.5 M. This can be determined by the salting-out effect. That is, as the ionic strength increases, the solubility of IBP decreases so that the sorption onto the medium can be enhanced [29]. Overall, MPPAC-Fe(3.8%) showed q_eq_ of 80.7–94.1 mg g^-1^, which was comparable to PPAC (76.3-94.7 mg g^-1^). Based on the total surface area, MPPAC-Fe(3.8%) had a higher sorption density (0.118–0.137 mg m^-2^) than PPAC (0.098–0.122 mg m^-2^).

The solution pH is a significant parameter in the sorption process, because it determines the charge of both adsorbent and adsorbate, and thereby governs the sorbent-sorbate electrostatic interaction [30]. Accordingly, pH plays a dominant role in determining the sorption capacities for all types of medium [16]. With a pK_a_ value of 4.91, when pH values are higher than pK_a_, the IBP molecules convert to anionic species, and almost all will be deprotonated at pH 7. Based on the calculated log D value (Table A in S1 File) of IBP at pH 4 and 7, this indicates that with a higher value of log D, the hydrophobicity of the molecules increases. Thus, the hydrophobicity of IBP is higher at a pH lower than the pK_a_.

The pH_PZC_ value (9.11) of MPPAC-Fe(3.8%) was lower than that (9.68) for PPAC (Fig B in S1 File.). Although Fe-impregnated AC had a lower pH_PZC_, both media showed a predominantly basic nature caused by delocalization of π electrons on the carbon plane [31]. However, as shown in the FTIR analysis, acidic oxygen functional groups formed on MPPACs, decreasing the pH_PZC_ value due to their acidic properties [25, 32].

The equilibrated adsorption capacities of IBP by PPAC, MPPAC-Fe(3.8%), and MPPAC-Fe(8.6%) were 111.7, 113.9, and 87.6 mg g^-1^ at pH 4, respectively, which were 1.23-1.35-fold higher than those at pH 7 [(Fig 4D and 4E)]. Similar to previous results, MPPAC-Fe(3.8%) had better adsorption capacities than the other media under both pH conditions. Moreover, the kinetic parameters, K_2_ (4.88–5.98×10^−3^ g mg^-1^ min^-1^) and v_0_ (2.72–4.08 mg g^-1^ min^-1^), were higher at pH 7 than at pH 4. Thus, under neutral conditions, a faster sorption rate was observed. In terms of kinetics, a neutral pH was ‘better’ than pH 4 due to the electrostatic interactions between anionic IBP and the positively charged surface of the media at pH lower than pH_PZC_ [33]. At pH 4, IBP exists mostly as a non-ionic species (89 mole %), restricting the favorable electrostatic interaction. Nevertheless, pH 4 showed a higher adsorption capacity than pH 7. As the main removal mechanism, the aromatic ring of IBP was positioned at the oxygen molecule of the C = O group as a donor-acceptor complex, as well as dispersive π-π interactions with the carbon surface. However, as a negatively charged species, IBP at pH 7 (55 mole %), it is difficult to have a molecular position due to the electrostatic interaction between the carboxylic group and the positively charged groups on the carbon surface.

### Isotherm

**Fig 5:**
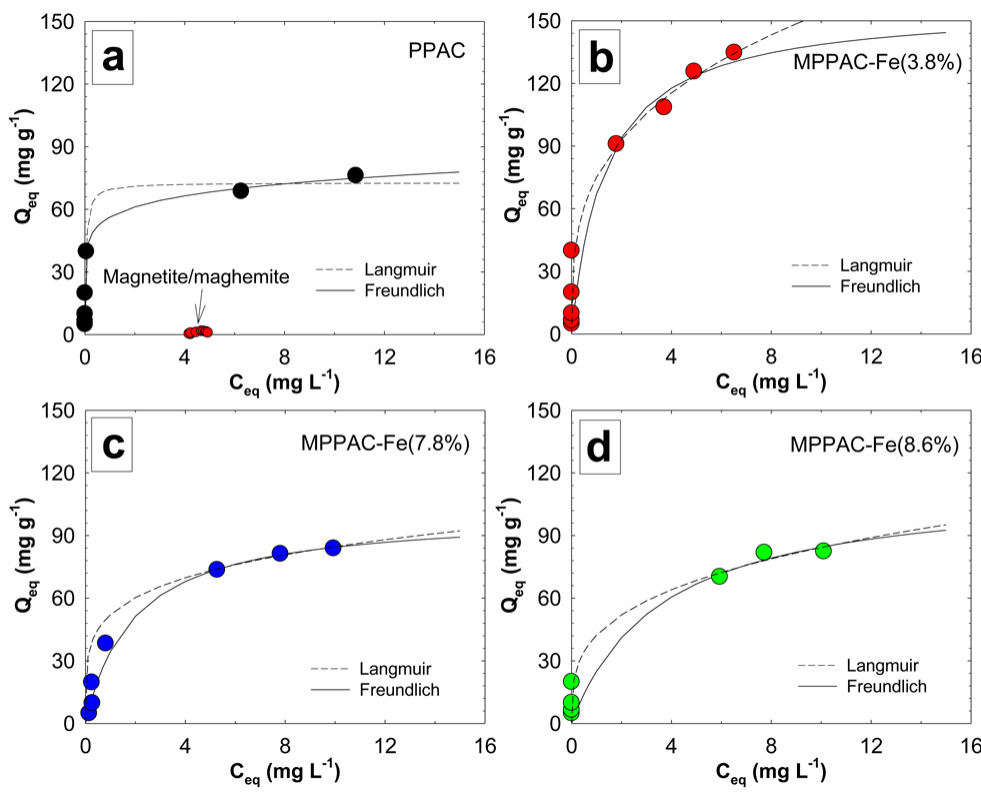
(a-d) Adsorption isotherms of IBP uptake by media at pH 7.0 and 0.1 M ionic strength, Langmuir (long dashed line) and Freundlich models (solid line).

Adsorption isotherm data were fitted to the Langmuir and Freundlich models (Fig 5) and were used to evaluate the Q_max_ of PPAC and MPPACs for IBP uptake. The equilibrium data and other calculated values are presented in Table 2. Based on the values of the determination coefficients (R^2^), the Freundlich model better fitted the data for PPAC and MPPAC-Fe(3.8%), whereas the Langmuir model was best MPPAC-Fe(7.8%) and -Fe(8.6%). As a function of adsorption strength, the values of 1/n for PPAC and MPPACs were 0.12-0.31, which were < 1, indicating that the adsorption is favorable under the conditions studied.

**Table 2:** Fitting parameters to the Freundlich and Langmuir models.

Model	Parameters	PPAC	MPPAC-Fe(3.8%)	MPPAC-Fe(7.8%)	MPPAC-Fe(8.6%)
Langmuir	K_L_ (g mg^-1^ min^-1^)	21.8	0.74	0.52	0.28
	Q_max_ (mg g^-1^)	72.7	157.3	100.6	114.7
	R^2^	0.989	0.947	0.993	0.837
Freundlich	K_F_ (g mg^-1^ min^-1^)	56.3	75.1	52.1	42.3
	1/n	0.12	0.31	0.21	0.3
	R^2^	0.997	0.978	0.977	0.78

MPPAC-Fe(3.8%) had the highest Q_max_ (157.3 mg g^-1^), which is about 2.2-fold higher than PPAC (72.7 mg g^-1^). Based on the total surface area, MPPAC-Fe(3.8%) had a sorption density 0.23 mg m^-2^, which is 2.5-fold higher than PPAC (0.094 mg m^-2^).

**Fig 6:**
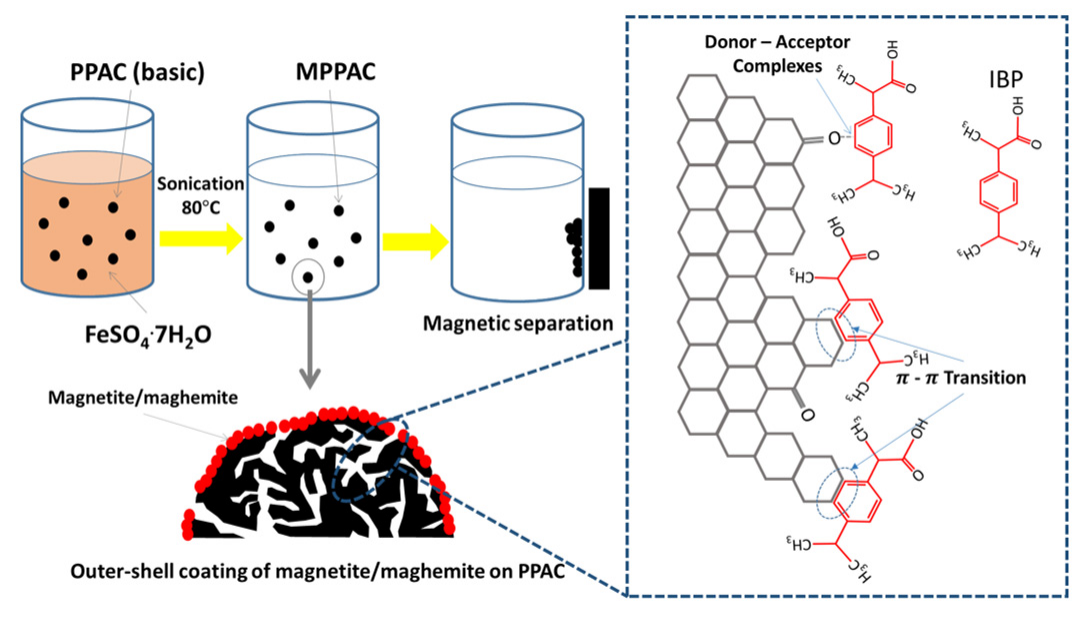
Synthesis route for MPPAC and possible IBP adsorption mechanism on activated carbon.

To verify the removal mechanism of IBP by PPAC and MPPACs, a sole magnetite/maghemite was synthesized with the same procedure as for the MPPACs, and its adsorption capacity was tested at 5 mg L^-1^ IBP. Adsorption tests showed that magnetite/maghemite had no sorption capacity. Thus, dispersive π-π interactions between the aromatic ring of IBP and hexagonal graphite of the activated carbon can be proposed to be the major removal mechanism of IBP by PPAC or MPPACs [34]. As shown with FTIR, the chemical properties of the pore surface for MPPACs differed from that of PPAC. The high sorption density of IBP for MPPAC-Fe(3.8%) may be linked to the C = O group created by the oxidation of the carbon surface during magnetite/maghemite impregnation. Guedidi *et al*. suggested that IBP was attracted mainly to the C = O surface group, where the carbonyl group serves as an electron donor, while the aromatic ring of IBP as the acceptor, leads to the formation of donor-acceptor complexes. However, as shown by the fact that MPPAC-Fe(3.8%) exhibited lower levels of C = O groups and a higher sorption capacity than MPPAC-Fe(8.6%), a slightly oxidized carbon surface may promote IBP removal because pore structures could be blocked when higher amounts of the oxygenated group are formed during the oxidation process [32]. As additional evidence, Kyzas *et al*. reported that the adsorption capacity of pharmaceutical compounds for 4 h-oxidized AC was 117 mg g^-1^, while 5 h of oxidation showed a decrease in adsorption capacity (91 mg g^-1^), although the total oxygen functional group increased twofold [35]. Fig 6 shows the overall synthesis route of MPPACs and a plausible IBP removal mechanism.

### Temperature effect and thermodynamic parameters

Temperature dependency for IBP uptake by MPPAC-Fe(3.8%) was studied using isotherm tests at pH 7. All isotherm data were fitted to the Langmuir and Freundlich models. As a result, both isotherm models provided the good fits (R^2^ = 0.98–0.99). Thermodynamic parameters were also calculated using the isotherm data. At various temperatures, the Q_max_ values obtained with the Langmuir model were similar, in the range of 127.5–131 mg g^-1^ (Fig C in S1 File). The Langmuir and Freundlich constants, K_L_ and K_F_, were also comparable, although adsorption affinity to IBP increased slightly at higher temperatures. This was also confirmed by the increase in ‘n,’ indicating higher adsorption affinity.

Based on the K_d_ values calculated at 50 mg g^-1^ sorption capacity, the estimated ΔG° were -1.69, -3.67, and -7.99 kJ moL^-1^ at 298, 308, and 318 K, respectively. Generally, the low range of ΔG° (0 to -20 kJ mol^-1^) can be defined as physi-sorption, while the high range (-80 to -400 kJ mol^-1^) can be interpreted as chemi-sorption [36]. Thus, IBP adsorption by MPPAC-Fe(3.8%) was a spontaneous physi-sorption process. The calculated ΔH° and ΔS° values were 6.35 kJ mol^-1^ and 21.6 J K^-1^ mol^-1^, respectively. Due to the positive value of ΔH°, the adsorption of IBP by MPPAC-Fe(3.8%) can be defined as an endothermic process. Although the positive value of ΔS° is indicative of the increase in disorder at the solid-solution interface due to the desorption of water, the ΔS° value for MPPAC-Fe(3.8%) was lower than other reference values (Table 3). This indicates that adsorption of IBP is less competitive with water molecules.

**Table 3:** Gibb’s free energy change, enthalpy change and entropy change for IBP adsorption.

Media	Kelvin (K)	Gibbs free energy, ΔG° (kJ mol^-1^)	Isosteric enthalpy, ΔH° (kJ mol^-1^)	Entrophy of adsorption, ΔS° (J mol^-1^ K^-1^)	Reference
Cork waste AC	298–313	-7.3	8.1	51.6	[32]
Clothes AC	298–328	-1.0	60.2	205.4	[37]
*Artemisia vulgaris* AC	298–318	-1.75 to -5.73	57.5	198.2	[38]
MPPAC-Fe(3.8%)	298–318	-1.69 to -7.99	6.35	21.61	This study

### Repeated adsorption

**Fig 7:**
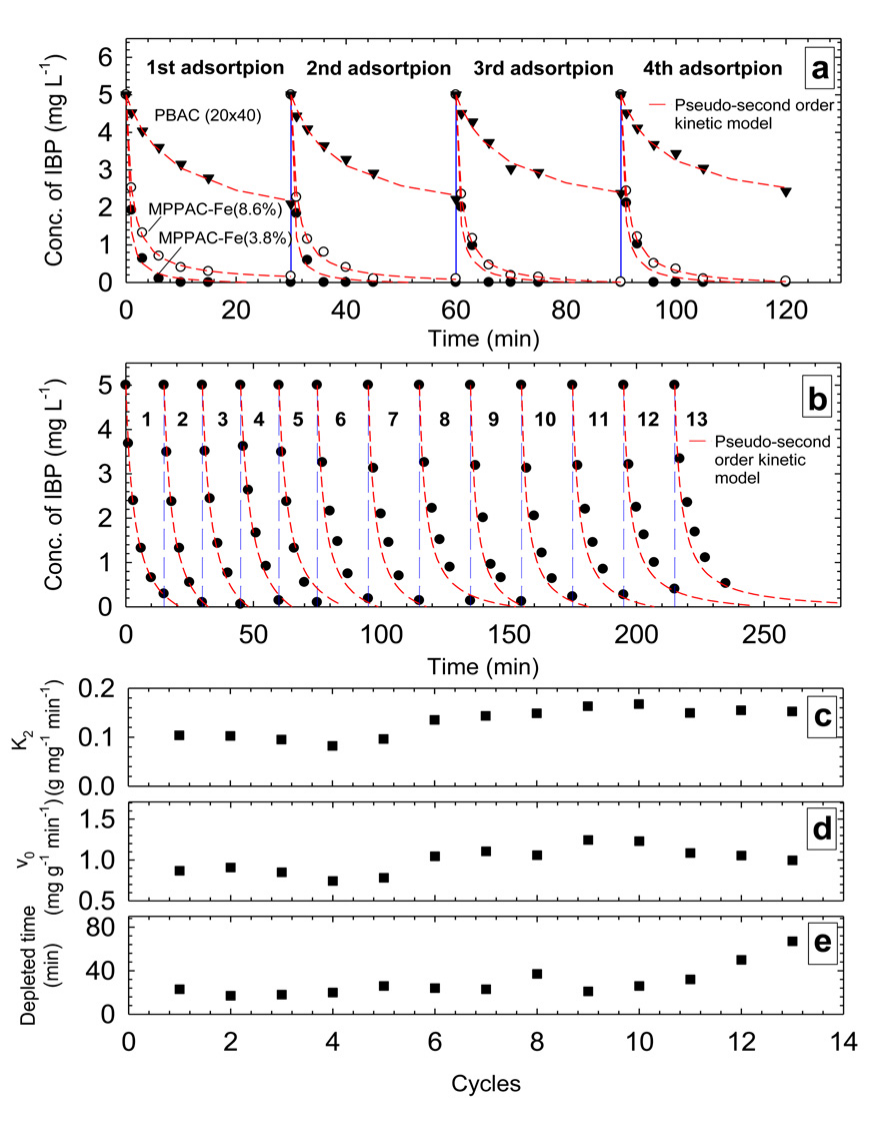
Repetitive removal of 5 mg L^-1^ IBP at pH 7.0 with 20 mg absorbent (a) 4 continuous cycles with MPPAC-Fe(3.8%), MPPAC-Fe(8.6%) and GAC, and (b) IBP removal using MPPAC-Fe(3.8%) with 13 successive cycles (Long-dashed lines denote pseudo-second order kinetic), (c-e) The adsorption rate (k_2_), initial adsorption rate (v_0_) and depleted time (min) was determined through pseudo second order fitting.

The first repeated removals of IBP were conducted to compare the rate of IBP uptake for each medium and cycle. Each cycle was operated for 30 min. Among the media, MPPAC-Fe(3.8%) had the highest removal speed for IBP [Fig 7A]. The kinetic results demonstrated that IBP was removed completely by MPPAC-Fe(3.8%) within 6 min, except for the first cycle, which required 10 min for completion. In the case of MPPAC-Fe(8.6%), all IBP was removed completely within 30 min during the second to fourth cycles, but about 0.16 mg L^-1^ IBP remained at 30 min for the first cycle. This can be explained by the establishment of Na^+^ layer on the media’s surface during the first cycle. PGAC did not remove IBP completely, and its removal efficiency decreased gradually from 58 to 51% over four cycles. Thus, PGAC and MPPACs showed different removal phenomena as the number of cycles increased. As shown in the kinetic results, the removal of IBP by PPAC was hindered by anionic Cl^-^ while MPPACs could have promoted removal by the Donnan effect as Na^+^ is accumulated on the surface of magnetite/maghemite. Through fitting data with the pseudo-second-order kinetic model, MPPAC-Fe(3.8%) had the highest K_2_ (0.76–1.16 g mg^-1^ min^-1^) and v_0_ (4.96–7.5 mg g^-1^ min^-1^), respectively. The kinetic comparison showed that K_2_ of MPPAC-Fe(3.8%) was about 1.9~2.8- and 9.1~15.8-fold higher than MPPAC-Fe(8.6%) and PGAC, respectively. For the case of v_0_, MPPAC-Fe(3.8%) was 1.8~2.5- and 24.6~35.4-fold higher than MPPAC-Fe(8.6%) and PGAC.

A second phase of repeated adsorption tests using MPPAC-Fe(3.8%) was conducted for 13 cycles with a shortened operational time (15 min). Although, except for the first cycle, IBP was almost depleted at 15 min in each cycle during the second to ninth cycles, a slower adsorption rate was observed compared with the first phase [Fig 7B]. The K_2_ and v_0_ obtained for 13 cycles were 0.1~0.18 g mg^-1^ min^-1^ and 0.8~1.3 mg g^-1^ min^-1^, respectively [Fig 7C and 7D]. Compared with the first phase, the reduction of these values occurred due to a shortened operation time, with which diffused IBP previously adsorbed can block the diffusion of IBP in subsequent cycles. Hypothetically, this happened due to the fact that the rate-determining step of IBP adsorption is pore diffusion, because most sorption sites are at micropores (< 2 nm). To prove this hypothesis, several kinetic models, such as the pseudo-first order, pseudo-second order, simple Elovich, power function, and parabolic diffusion, were applied to find the values of R^2^ through fitting kinetic data (Table C in S1 File). As a result, the parabolic diffusion had the highest R^2^ for all cases, suggesting that the adsorption of IBP was diffusion-controlled (Table D in S1 File) [39, 40]. The depletion time was also obtained by fitting data with the pseudo-second order kinetic model (Fig 7E). As K_2_ and v_0_ decreased, the depletion time of IBP increased, to 36-67 min in the 10th-13th cycles.

### Thermal regeneration and re-adsorption

**Fig 8:**
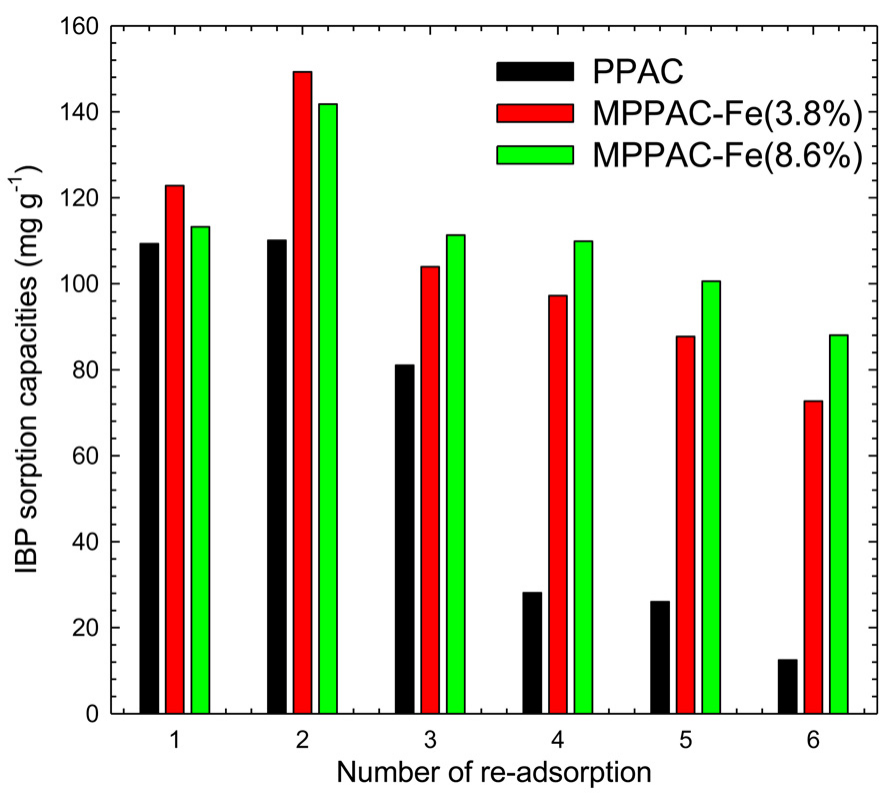
Adsorption capacities of saturated adsorbents after 6 cycles of thermal regeneration at 623.15 K.

Thermal regeneration and re-adsorption tests were conducted six times (Fig 8). MPPACs showed better re-adsorption capability than PPAC. For each cycle, PPAC and MPPACs showed different adsorption patterns. In the case of PPAC, the sorption capacity was reduced sharply after the second cycle. At the sixth cycle, the sorption capacity was only 13 mg g^-1^. Both MPPAC-Fe(3.8%) and MPPAC-Fe(8.6%) showed increases in sorption capacities at the second cycle, but the sorption capacities decreased gradually to 70 and 86 mg g^-1^ at the sixth cycle, respectively, which were 41 and 22% reductions compared with the first sorption values.

Based on the results obtained, we suggest that the magnetite/maghemite have thermal catalytic effects to reduce the desorption energy [41, 42]. Moreover, the low-temperature desorption of IBP could indicate that the adsorption was linked to physi-sorption rather than chemi-sorption [43]. The reduction of adsorption capacities after several cycles is due to incomplete desorption of IBP adsorbed in micropores or exhaustion of functional groups. However, further investigations should be performed to determine the optimal regeneration temperature without damaging the carbon pore structures. The magnetic properties of MPPAC were not changed even after six cycles of regeneration. Other regeneration method such as using acid-base [44] for desorption would be unfavorable due to dissolution of magnetite low pH condition.

## Conclusions

Via an economic and scalable preparation method, we successfully synthesized MPPAC where magnetite/maghemite was deposited homogeneously on the outer surface of PPAC. Based on the results of FTIR, it was found that Fe impregnation using ultrasound resulted in oxidation of the surface carbon of PPAC, to form phenolic and C = O groups. Remarkably, isotherm results showed that MPPAC-Fe(3.8%) had about a 2.2-fold higher Q_max_ (157.3 mg g^-1^) and a 2.5-fold higher sorption density (0.23 mg m^-2^) than PPAC. Based on the overall results, the major mechanism of IBP removal was found to be donor-acceptor complexes, with a C = O group and dispersive π-π interaction with a carbon surface. With the repeated adsorption tests, it was found that pore diffusion was the rate-determining step and MPPAC-Fe(3.8%) had a much faster removal capacity than MPPAC-Fe(8.6%) and PGAC. Magnetized activated carbon showed better regeneration than PPAC due to a thermal catalytic effect of magnetite/maghemite at a low thermal temperature.

## Supporting Information

Supporting Information contains the methodology regarding various instrumental analysis, physical and chemical characteristics of IBP, kinetic and isotherm models, and kinetic values for ionic strength effect. Detailed description of data on pore characteristics, adsorption kinetics, the relationship between Fe contents with pore characteristics, and temperature effect on adsorption isotherms.

## Supporting Information

S1 File Media characterizations and adsorption parameters. Physicochemical properties of ibuprofen **(Table A)**. Comparisons of adsorption kinetics of IBP uptake by media at different ionic strength **(Table B)**. Linear plots and equation for 5 different kinetic models **(Table C)**. Determination Coefficient (R^2^) of GAC, MPPAC-Fe(3.6%) and MPPAC-Fe(8.6%) through data fitting with different kinetic models. Through phase 1, all adsorbent were continuously adsorb for 4 times while in 2^nd^ phase, only MPPAC-Fe(3.8%) were repeated 14 times **(Table D).** Relationship between Fe contents with pore characteristics results **(Fig A).** pH_ZPC_ of PPAC and MPPAC–Fe(3.8%) with deionized water and 0.1 M NaCl **(Fig B).** Temperature effect on adsorption isotherm of IBP: MPPAC-Fe(3.8%) at pH 7. Line curve: Langmuir model; Long dashed curve: Freundlich model **(Fig C).**
(DOCX)
