# Supplementary material for: Enhanced Recyclable Magnetized Palm Shell Waste-Based Powdered Activated Carbon for the Removal of Ibuprofen: Insights for Kinetics and Mechanisms
Source: PLoS One. 2015 Oct 23;10(10):e0141013. doi: 10.1371/journal.pone.0141013 (PMC4619863; doi:10.1371/journal.pone.0141013)
Supplement: S1 File — Physicochemical properties of ibuprofen (Table A). Comparisons of adsorption kinetics of IBP uptake by media at different ionic strength (Table B). Linear plots and equation for 5 different kinetic models (Table C). Determination Coefficient (R2) of GAC, MPPAC-Fe(3.6%) and MPPAC-Fe(8.6%) through data fitting with different kinetic models. Through phase 1, all adsorbent were continuously adsorb for 4 times while in 2nd phase, only MPPAC-Fe(3.8%) were repeated 14 times (Table D). Relationship between Fe contents with pore characteristics results (Fig A). pHZPC of PPAC and MPPAC–Fe(3.8%) with deionized water and 0.1 M NaCl (Fig B). Temperature effect on adsorption isotherm of IBP: MPPAC-Fe(3.8%) at pH 7. Line curve: Langmuir model; Long dashed curve: Freundlich model (Fig C). (DOCX) [file pone.0141013.s001.docx]

**Supporting information**

## Physicochemical characterization

The prepared media was analyzed by X-ray diffraction (XRD, Bruker D8 advance X-ray powder diffractometer with Cu Kα radiation λ=1.5406Å) in the region from 10° to 80° of 2θ.

The resulting morphologies of PPAC and MPPAC were examined by field emission scanning electron microscopy (FESEM, Hitachi S-4300) and energy dispersed spectroscopy (EDS, Horiba EX-200).

Nitrogen adsorption–desorption isotherms were performed at 77 K by TriStar II 3020 (Micrometrics®, USA). The BET surface area and pore size distribution were determined using Brunauer Emmett Teller (BET) model and pore size distributions (width range from 17 to 3,000 Å) were determined by the Barrett-Joyner-Halenda (BJH) method ([Barrett et al., 1951](#_ENREF_3)).

The surface properties of all samples were determined by Fourier transform infrared (FTIR) using Perkin Elmer (FTIR-Spectrum 400). Samples were prepared by combining into a KBr pellet and the infrared spectrum from 4000 to 450 cm^-1^ was obtained. The magnetism of materials was measured by an alterating gradient magnetometer (AGM), micromag 2900 AGM.

The pH of zero-charge (pH_pzc_) for PPAC and MPPAC–Fe(3.8%) were determined by pH drift method ([Franz et al., 2000](#_ENREF_15)). Samples of 0.02 g were added to a 10 mL 0.1M NaCl solution at different pH values (2, 4, 6, 8 and 10) and shaked for 48 h. The pH of the resulting suspension was measured after filtration and the pH_pzc_ values were determined by plotting an initial pH vs final pH.

## Batch tests of kinetics and isotherm

The experimental data of kinetics were fit using pseudo-second order kinetic model. The linearized form of pseudo-second order Eq. (1) can be written as,

$\frac{t}{q_{t}}=\frac{1}{k_{2}q_{eq}^{2}}+\frac{t}{q_{eq}}$ (1)

Where k_2_ is the pseudo-second order rate constant (g mg^-1^ min^-1^), q_eq_ and q_t_ are the adsorbate uptake (mg g^-1^) at equilibrium and time t, respectively. The values of q_eq_ and k_2_ can be estimated from the slope and the intercept, respectively, of the plot (t/q_t_) versus t. From the equation, the product k_2_ q_eq_^2^ (v_0_, mg g^-1^ min^-1^) represents the initial adsorption rate.

The adsorption capacity of IBP at equilibrium, q_eq_ was calculated by the Eq. (1),

$q_{eq}=\frac{(C_{eq}-C_{0})\times V}{m}$ (2)

where C_o_ and C_eq_ are the initial and equilibrated IBP concentrations (mg L^-1^), respectively, V is the solution volume, and m is the mass of the dried MPPAC (g).

When adsorption is held to monolayer and uniform surface, the Langmuir model fits the data of isotherm. The maximum adsorption capacity will be achieved when all sorption sites are saturated. The linear form of Langmuir model Eq. (3) can be expressed as:

$q_{eq}=\frac{Q_{\max}K_{L}C_{eq}}{1+K_{L}C_{eq}}$ (3)

where q_eq_ is the amount of solute adsorbed per unit weight of adsorbent (mg g^-1^), C_eq_ is the equilibrium concentration of solute in the bulk solution (mg L^-1^), Q_max_ is the maximum adsorption capacity (mg g^-1^), and K_L_ is the Langmuir constant related to the energy of adsorption.

Assuming that the adsorption is held onto the heterogeneous surface of adsorbent, the Freundlich model better fit the isotherm data. In the Freundlich model, chemisorption and physisorption are pertinent to monolayer and multilayer adsorption, respectively. The linear form of Freundlich equation is expressed as:

$\log q_{eq}=\log K_{F}+\frac{1}{n}\log C_{eq}$ (4)

Where K_F_ and n are Freundlich isotherm constants related to adsorption capacity and adsorption intensity, respectively.

**Table A. Physicochemical properties of ibuprofen**

| **Physical and Chemical Properties** | | **Ibuprofen (IBP)** |
| --- | --- | --- |
| Molecular Structure | | **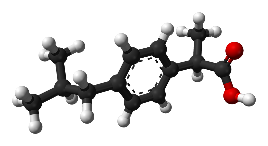** |
| pK_a_ | | 4.91 |
| Abs (nm) | | 220 |
| Solubility in water (mg L^-1^) | | 21 |
| Log K_ow_ | | 2.48 |
| Molecular Dimension (nm) | | 1.00 × 0.60 |
| Log D | At pH 4 | 2.430 |
|  | At pH 7 | 0.386 |

**Table B. Comparisons of adsorption kinetics of IBP uptake by media at different ionic strength**

|  | Ionic Strength (M) | Media | | | |
| --- | --- | --- | --- | --- | --- |
|  |  | PPAC | MPPAC–Fe(3.8%) | MPPAC–Fe(7.8%) | MPPAC–Fe(8.6%) |
| *q_eq_*  (mg g^-1^) | 0.0 | 76.3 | 80.7 | 66.8 | 67.1 |
|  | 0.1 | 82.6 | 90.7 | 69.3 | 71.4 |
|  | 0.5 | 94.7 | 94.1 | 78.3 | 80.2 |
| *k_2_*  (g mg^-1^ min^-1^) | 0.0 | 0.000742 | 0.000489 | 0.000499 | 0.000577 |
|  | 0.1 | 0.000598 | 0.000488 | 0.000577 | 0.000535 |
|  | 0.5 | 0.000428 | 0.000409 | 0.000446 | 0.000396 |
| *v_0_*  (mg g^-1^ min^-1^) | 0.0 | 4.33 | 3.18 | 2.23 | 2.59 |
|  | 0.1 | 4.08 | 4.01 | 2.77 | 2.73 |
|  | 0.5 | 3.84 | 3.62 | 2.73 | 2.55 |
| R^2^ | 0.0 | 0.996 | 0.994 | 0.990 | 0.991 |
|  | 0.1 | 0.998 | 0.999 | 0.996 | 0.996 |
|  | 0.5 | 0.997 | 0.996 | 0.993 | 0.992 |

**Table C. Linear plots and equation for 5 different kinetic models**

| Kinetic Models | Linear plots | Equations |
| --- | --- | --- |
| Pseudo-first order | $\ln(q_{eq}-q_{t})$*vs. t* | $\ln(q_{eq}-q_{t})=\ln(q_{eq})-k_{1}t$ |
| Pseudo-second order | $t/q_{eq}$ *vs. t* | $\frac{t}{q_{t}}=\frac{1}{v_{0}}+\frac{1}{q_{eq}}t$ |
| Power function | $\ln(q_{t})$vs. $\ln(t)$ | $\ln(q_{t})=\ln a+b\ln(t)$ |
| Parabolic diffusion | $q_{1}/q_{eq}/t$ vs $t^{-0.5}$ | $\frac{q_{t}}{q_{eq}t}=k_{diff}t^{-0.5}+a$ |
| Simplified Elovich | $q_{1}$vs $\ln(t)$ | $q_{1}=a\ln(t)+b$ |

**S4 Table. Determination Coefficient (R^2^) of GAC, MPPAC-Fe(3.6%) and MPPAC-Fe(8.6%) through data fitting with different kinetic models. Through phase 1, all adsorbent were continuously adsorb for 4 times while in 2^nd^ phase, only MPPAC-Fe(3.8%) were repeated 14 times**

| **1^st^ phase continuous sorption tests** | | | | | | | | | | | | | | | | | | | | | | | | | |
| --- | --- | --- | --- | --- | --- | --- | --- | --- | --- | --- | --- | --- | --- | --- | --- | --- | --- | --- | --- | --- | --- | --- | --- | --- | --- |
| Kinetic  models | GAC | | | | | | | | MPPAC-Fe(3.6%) | | | | | | | | | MPPAC-Fe(8.6%) | | | | | | | |
|  | 1st | | 2nd | | 3rd | | 4th | | | 1st | | 2nd | | 3rd | | 4th | | 1st | | 2nd | | 3rd | | 4th | |
| Pseudo-second | 0.983 | | 0.971 | | 0.970 | | 0.977 | | | 0.999 | | 0.999 | | 0.999 | | 0.999 | | 0.999 | | 0.999 | | 0.999 | | 0.999 | |
| Pseudo-first | 0.986 | | 0.989 | | 0.943 | | 0.985 | | | 0.580 | | 0.489 | | 0.494 | | 0.496 | | 0.779 | | 0.752 | | 0.749 | | 0.764 | |
| Power function | 0.991 | | 0.998 | | 0.964 | | 0.994 | | | 0.767 | | 0.747 | | 0.796 | | 0.794 | | 0.876 | | 0.906 | | 0.866 | | 0.872 | |
| Simplified Elovich | 0.969 | | 0.950 | | 0.942 | | 0.963 | | | 0.793 | | 0.767 | | 0.806 | | 0.806 | | 0.918 | | 0.938 | | 0.903 | | 0.913 | |
| Parabolic Diffusion | 0.985 | | 0.998 | | 0.966 | | 0.995 | | | 0.996 | | 0.996 | | 0.995 | | 0.996 | | 0.998 | | 0.995 | | 0.998 | | 0.998 | |
| **2^nd^ phase continuous sorption tests** | | | | | | | | | | | | | | | | | | | | | | | | | |
| Kinetic models | MPPAC-Fe(3.8%) | | | | | | | | | | | | | | | | | | | | | | | | |
|  | 1st | 2nd | | 3rd | | 4th | | 5th | | | 6th | | 7th | | 8th | | 9th | | 10th | | 11th | | 12th | | 13th |
| Pseudo-second | 0.997 | 0.997 | | 0.992 | | 0.989 | | 0.994 | | | 0.995 | | 0.994 | | 0.995 | | 0.995 | | 0.998 | | 0.994 | | 0.993 | | 0.994 |
| Pseudo-first | 0.929 | 0.951 | | 0.965 | | 0.974 | | 0.965 | | | 0.964 | | 0.965 | | 0.960 | | 0.921 | | 0.943 | | 0.960 | | 0.963 | | 0.961 |
| Power function | 0.951 | 0.974 | | 0.980 | | 0.987 | | 0.982 | | | 0.987 | | 0.993 | | 0.985 | | 0.948 | | 0.974 | | 0.987 | | 0.991 | | 0.986 |
| Simplified Elovich | 0.986 | 0.989 | | 0.987 | | 0.983 | | 0.988 | | | 0.997 | | 0.996 | | 0.995 | | 0.973 | | 0.992 | | 0.993 | | 0.994 | | 0.994 |
| Parabolic Diffusion | 0.965 | 0.991 | | 0.993 | | 0.994 | | 0.993 | | | 0.999 | | 0.999 | | 0.999 | | 0.994 | | 0.999 | | 0.999 | | 0.999 | | 0.999 |

**Figure A. Relationship between Fe contents with pore characteristics results.**

**Figure B. pH_ZPC_ of PPAC and MPPAC–Fe(3.8%) with deionized water and 0.1 M NaCl**

**Figure C. Temperature effect on adsorption isotherm of IBP: MPPAC-Fe(3.8%) at pH 7. Line curve: Langmuir model; Long dashed curve: Freundlich model.**
